# Potential impact of climate change on the reproductive success of grayling ( *Thymallus thymallus* )

**DOI:** 10.1111/jfb.70160

**Published:** 2025-08-14

**Authors:** Simon Rosenau, Alexander Charles Mott, Christian Lodder, Jens Tetens

**Affiliations:** ^1^ Department of Animal Sciences University of Goettingen Göttingen Germany; ^2^ Center for Integrated Breeding Research University of Goettingen Göttingen Germany

**Keywords:** aquaculture, grayling stocking, incubation temperature, species conservation, water temperature, wild population

## Abstract

The European grayling is a salmonid species experiencing a sharp population decline, and conservation measures have been initiated through artificial propagation. As the species is not commonly cultivated in aquaculture, there is little information on their optimal reproduction conditions. Therefore, this study has combined a two‐pronged approach of examining the temperature of the natural spawning area during the spawning season, while also experimentally testing four different incubation temperatures under standardized aquaculture conditions. Nine egg clutches from pond‐reared females were fertilized with a sperm mix from 10 wild grayling. Eggs were divided into four treatment groups and incubated at 6, 8, 10 and 12°C. Hatched larvae were then collected and examined for body weight, yolk‐sac weight and length. Both the temperature of the natural habitat and the physiological parameters of the larvae indicate that 6 and 8°C are the optimal hatching temperatures for grayling reproduction. The hatching rate was highest at 8 and 6°C, at 80.35 ± 6.30% and 76.87 ± 8.01%, respectively, although it gradually decreased with increasing temperature. The impact of warmer water temperatures hindered survival rates, resulting in a significant decrease in hatching rates at 10°C (68.46 ± 13.85%) and 12°C (45.54 ± 27.14%).

## 1 INTRODUCTION

Since the mid‐1980s, the European grayling (*Thymallus thymallus*) populations have declined in Germany on average by over 90% (Laves, 2019). Meanwhile, grayling in general are considered to be a threatened fish species with rapidly declining population (BAFU, 2022; Gum et al., 2003; Uiblein et al., 2001). In most German and Austrian federal states, the grayling is now considered a critically endangered fish species (Didenko et al., 2018; Freyhof et al., 2023; Kodela et al., 2023; Laves, 2011; Spindler, 1997). Projections indicate that the potential habitat ranges of these species could decline dramatically, with reductions of up to 75% for grayling. With regard to the European biodiversity strategy for 2030 (European Commission, 2021), the conservation status of the grayling population is currently classified as unfavourable‐insufficient with a continuing deteriorating trend (BfN, 2023).

The reasons for endangerment are diverse, but degradation and habitat loss, linked primarily to human activities, play a key role. Due to increased soil erosion through intensive mechanical tillage and meltwater, or heavy rainfall events, large amounts of fine sediments enter the water bodies (NLWKN, 2011; Reckendorfer et al., 2019; Riley et al., 2006). In addition, urbanization and the associated canalization of river systems significantly contribute to the loss of grayling habitat (Čivas & Kesminas, 2011; Hertig, 2006). Dam constructions also pose a major threat by blocking migration to spawning habitats (Ovidio & Philippart, 2002) and inhibiting gene flow between populations. This can result in genetic drift and loss of variation in grayling populations (Swatdipong et al., 2010) but also blocking the longitudinal transfer of spawning sediment (Hauer et al., 2011).

The alteration of river channels and the added stress of water pollution are also detrimental to grayling survival (Opinion et al., 2020), which depends on clean, well‐oxygenated waters (Blohm et al., 1994) with specific substrate conditions that are essential for spawning (Hauer et al., 2013). Schmall (2009) identifies the water flow as a major driver for reproductive success. Further factors include severe bird predation on grayling (Avramović et al., 2025; Jepsen et al., 2018) and water temperature, which influence spawning migration and timing by acting as a physiological trigger. Increase in daily maximum temperatures can also initiate upstream movements and stimulate reproductive behaviours such as territoriality, courtship and spawning (Schmall, 2009). The shifts in temperature and precipitation patterns exacerbated by climate change are impacting the availability of suitable habitats for grayling (Pletterbauer et al., 2016).

Conservation efforts, such as the construction of artificial spawning grounds and habitat restoration, have been implemented (Nykänen et al., 2001; Watz et al., 2020; Zeh & Dönni, 1994) alongside fishing regulations and the establishment of protected areas to support grayling populations (Cove et al., 2019). Additionally, public awareness and education campaigns aim to promote responsible fishing practices and the importance of preserving grayling habitats (Cove et al., 2019). However, such measures are unlikely to offset the effects of climate change and the associated increase in water temperatures (Ficklin et al., 2023), which could have a significant impact on grayling reproduction in rivers.

For some European rivers it is predicted that water temperatures will increase by up to 3.5°C until the end of the century (Hardenbicker et al., 2017; Michel et al., 2022). In addition, local fishing clubs are trying to improve the grayling population with stocking measures or through reintroduction programmes for grayling (Turek et al., 2012; Turek et al., 2018). Avramović et al. (2024) have shown that stocking with fry can effectively support the recovery of natural populations. However, these stocking programmes heavily rely on artificial reproduction, which as indicated by the relatively low numbers of grayling stocked in recent surveys (Pagel & Arlinghaus, 2016) appears to be limited in practice. Furthermore, adapted stock fish can be hard to find or even potentially unavailable. This is due to grayling reproduction in aquaculture not being sufficiently tested and the limited availability of literature on the subject (Kodela et al., 2023). Although several publications have addressed grayling stocking material production under controlled conditions (Frunza et al., 2023; Lahnsteiner & Kletzl, 2014; Luczynski et al., 1986; Szmyt et al., 2012; Szmyt et al., 2015; Szmyt et al., 2020), it is important to note that this practice remains largely experimental, with a lack of consistent data on its success and no established guidelines for its implementation. Here, the field of reproduction is a vast reservoir of possibilities that have yet to be explored for grayling.

Successful reproduction depends on specific environmental conditions, including water temperature, oxygen availability and minimal sedimentation, which can be disrupted by habitat degradation and anthropogenic influences, as described previously. In aquaculture, these factors are strictly controlled to optimize egg survival, with stable water parameters and the use of specialized incubation systems ensuring high hatch rates and larval viability.

The aim of the study was to investigate the effect of four different incubation temperatures from 6 to 12°C on the hatching rate of grayling. We hypothesize that grayling hatching rates will be affected by incubation temperatures, which play a key role in determining hatching success. Higher temperatures are anticipated to decrease success, simulating the negative impact of climate change on grayling reproduction. Additionally, the water temperature of the grayling population's natural habitat was measured over the reproductive period. This was then used as the baseline for a comparative incubation experiment. The results of this study will provide information on optimal reproduction temperatures for use in aquaculture, as well as provide further information on the effects of increased water temperatures, linked to climate change, on grayling reproduction and their adaptive capacity to heat stress.

## 2 MATERIALS AND METHODS

### 2.1 Reproduction conditions

Three‐year‐old female grayling were reared and kept in flow‐through ponds with a water flow of ~5 L/s at the experimental farm of the University of Göttingen in Relliehausen (Dassel, Germany). The grayling population is a first‐generation aquaculture stock and was obtained from a wild stock population of the River Ilme, the same river that supplies the farm in Relliehausen. The fish farm is within 12 km of the river section used for natural spawning. According to a study from Köbsch et al. (2019), the wild population has not been altered by stocking with other grayling and represents an autochthonous population. The aquaculture stock was fed with a commercial trout feed (Biomar, Brande, Denmark; Table 1). Male fish from this wild population were caught by electrofishing and were used to crossbreed into the aquaculture population.

**TABLE 1:** Nutrient composition and energy content of commercial trout feed (EFICO Alpha 714, Biomar).

Parameter	Unit	Value
Crude protein	%	40.0–43.0
Crude fat	%	14.0–17.0
Carbohydrates (NFE)	%	26.0–29.0
Crude fibre	%	3.3–6.6
Ash	%	4.0–7.0
Total phosphor (P)	%	0.9
Gross energy	MJ/kg	19.0–22.0
Digestible energy	MJ/kg	15.7

Abbreviation: NFE, nitrogen‐free extract.

**FIGURE 1:**
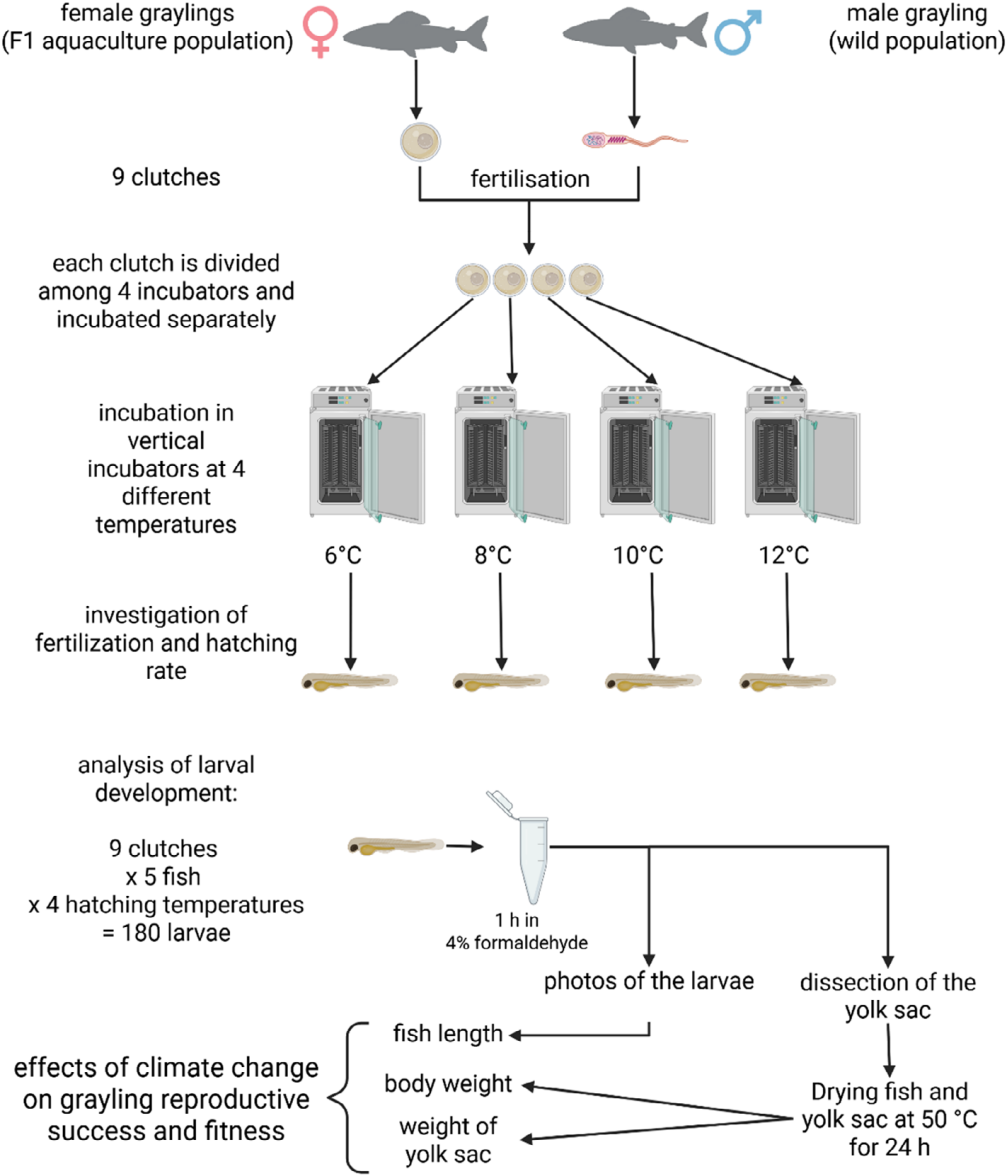
Graphical abstract of the reproduction and incubation conditions as well as sample collection.

A graphical abstract for this part of the methods is shown in Figure 1. At the end of March 2023, reproduction occurred on a single day. The parental fish were anaesthetized with clove oil (0.4 g/L), and female and male gametes were obtained by stripping and then used for reproduction. The sperm was directly collected from the genital papilla using a glass pipette to prevent any contact with water. A mix of sperm from 10 individual males was used to fertilize 9 individual clutches of eggs, each originating from a single female, with water added to initiate fertilization. The eggs were then water hardened for 1 h and then rinsed with clear water. All clutches were packed into individual glass containers, placed in a Styropor box for temperature maintenance and then brought to the experimental facility of the Department of Animal Sciences in Göttingen (45 km distance). There, the clutches were quartered by volume and placed in four separate, identical vertical incubators (VECO AG, Horgen, Switzerland) with four different incubation temperatures (6, 8, 10 and 12°C water temperature), with the clutches distributed across all incubators in the same order. Water temperature was measured twice a day using a digital thermometer, testo 925 thermometer (Testo SE & Co. KGaA, Titisee‐Neustadt, Germany), and degree‐days (DD) were calculated as follows:

(1) $$\mathrm{DD}=\sum \left(T_{i}^{\text{mean}}-T_{0}\right)$$

where $T_{i}^{\text{mean}}$ represents the mean daily temperature for every point in time from $T_{1}$ to $T_{n}$ and $T_{0}$ is the threshold temperature (describes the temperature below which growth or development of the embryo is effectively zero). A value of zero is applied to $T_{0}$ for salmonid species like grayling (Chezik et al., 2014).

All incubators were disinfected with formalin 1 week before the trial started. The incubators were connected to a separate recirculation system which was equipped with a Titan 1500 cooling unit (Aqua Medic GmbH, Bissendorf, Germany). Minimal water exchange of the system was ensured by tap water. Dead eggs were removed and counted 24 h after fertilization, and this was repeated after the eyed stage was visible. At the beginning of hatching, dead larvae were also counted. After 99.9% of the larvae had hatched, all healthy larvae were counted. Malformed larvae and unhatched eggs were counted as mortality.

### 2.2 Monitoring the River Ilme water temperature in the natural spawning area of grayling

The area of the River Ilme from which the grayling originated was also investigated to assess the natural incubation temperature of the wild grayling eggs. In the river section where natural spawning occurs, an EasyLog temperature logger (Lascar Electronics, Whiteparish, England) was installed ~12 km downstream from the aquaculture facility (coordinates: 51.80754930496516, 9.856600955261928), recording data every 30 min. Temperature monitoring began at the onset of reproduction in the experimental fish and continued until hatching was completed in all grayling, including those in the 6°C treatment group, which represented the longest incubation period (30 March 2023 to 12 May 2023).

### 2.3 Sampling of larvae

The sampling method was adapted from Ojanguren and Braña (2003) with modifications: to assess the developmental stage and vitality of the larvae, five healthy larvae from each clutch were killed by an overdose of 2‐phenoxyethanol after about 50% of the experimental group had hatched and then placed in 4% formalin for 1 h to harden the body of the larvae for dissection. The larvae were then placed in a Petri dish with a millimetre scale, and images were obtained using a Nikon D7200 reflex camera and a Nikon AF‐S Micro NIKKOR 40‐mm 1:2.8 G lens (Nikon, Minato, Japan), mounted on a tripod with a fixed distance to the fish. The images were later used to determine the length using ImageJ software (version 1.54d). After the images were obtained, the yolk sac was dissected from the body (Figure S1) and placed in separate tubes. The body parts were then dried at 50°C for 24 h to remove water content and obtain an accurate measurement of their dry weight, ensuring precise and consistent results. Dry weight of the fish body and the appendant yolk sac was measured to the nearest of 0.1 mg using a Quintix 224‐1S scale (Sartorius, Göttingen, Germany).

### 2.4 Statistical analysis

The statistical programme R (version 4.1.1) was used for data analysis. The data were visualized using ggplot2 package (version 3.4.2) (Wickham, 2016). LOESS (locally weighted scatterplot smoothing) was applied to the water temperature data from the River Ilme to capture and highlight trends without being influenced by short‐term fluctuations. Normality was visually checked using a quantile–quantile plot (Aldor‐Noiman et al., 2013). Normally distributed data were analysed using one‐way analysis of variance (ANOVA) with subsequent Tukey's honestly significance difference test (Barros, 2013). Data that did not follow a normal distribution were analysed using the Friedman test, followed by the Friedman–Nemenyi post hoc test. A significance level of 0.05 was used for all statistical analyses.

## 3 RESULTS

### 3.1 Egg hatching and survival

The temperatures of the four incubators over the course of the experiment were as follows: 5.80 ± 0.10°C (6°C), 8.05 ± 0.19°C (8°C), 10.12 ± 0.23°C (10°C) and 12.11 ± 0.15°C (12°C). Each treatment group contained between 307 and 1536 eggs, with total clutch sizes ranging from 1264 to 5904 eggs across the nine different clutches (Table S1). Eyeing stage was reached after 96–108 DD, and the first larvae hatched between 152 and 168 DD. At 228–264 DD the hatching was complete. Higher water temperatures accelerated embryonic development, resulting in an earlier onset of eying, as well as an earlier initiation and completion of hatching.

**FIGURE 2:**
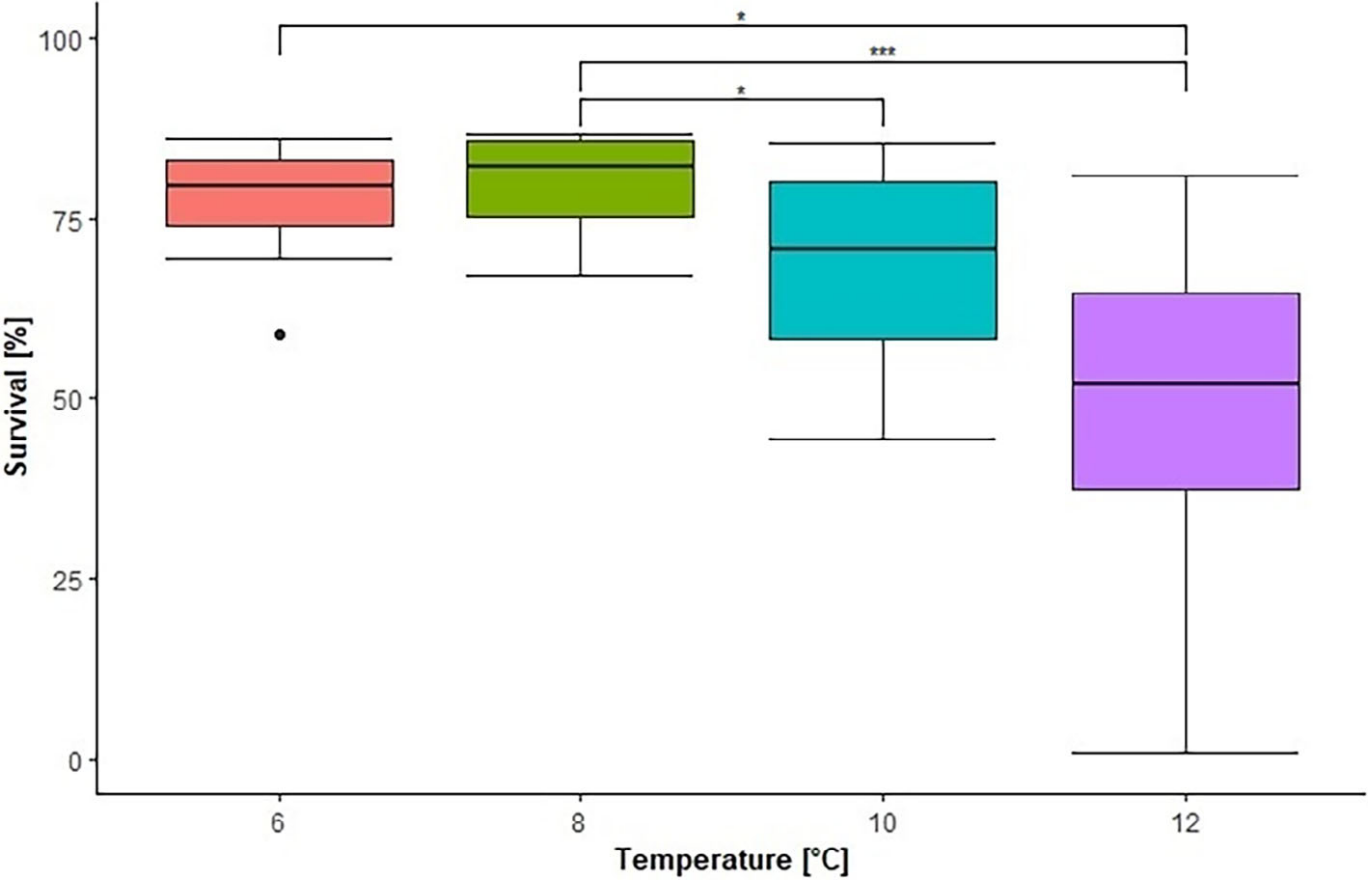
Survival rate of nine grayling clutches divided into four different incubation temperatures. *p < 0.05, **p < 0.01, and ***p < 0.001.

The 8°C treatment group had the highest survival rate of 80.35 ± 6.30%, closely followed by the 6°C group with 76.87 ± 8.01% (Figure 2). With increasing water temperatures, the survival rate gradually decreased to 68.46 ± 13.85% in the 10°C group and to 45.54 ± 27.14% in the 12°C group. The Friedman test revealed a significant difference among the treatment groups (*p* < 0.05). Subsequent post hoc analyses using the Friedman–Nemenyi test identified significant differences between the 6 and 12°C groups (*p* < 0.05), 8 and 10°C group (*p* < 0.05) and 8 and 12°C groups (*p* < 0.001).

In addition, a visual observation during the experiment indicated that with increasing water temperature, eggs were increasingly infested with fungus (*Saprolegnia* spp.). Larval mortality, calculated as the proportion of dead larvae relative to the total number of eggs within one egg clutch, was low across all groups, averaging between 2.45 ± 2.14% and 5.82 ± 2.33% (Table S2).

The standard deviation in the 12°C group was relatively high, indicating that whereas some clutches have high survival rates, others experienced significant egg loss (Table S3). Although all fish were fed a commercial trout diet without carotenoids (Biomar), one clutch had a strong red colouration (clutch number 8).

### 3.2 Water temperature of the natural habitat in the River Ilme

**FIGURE 3:**
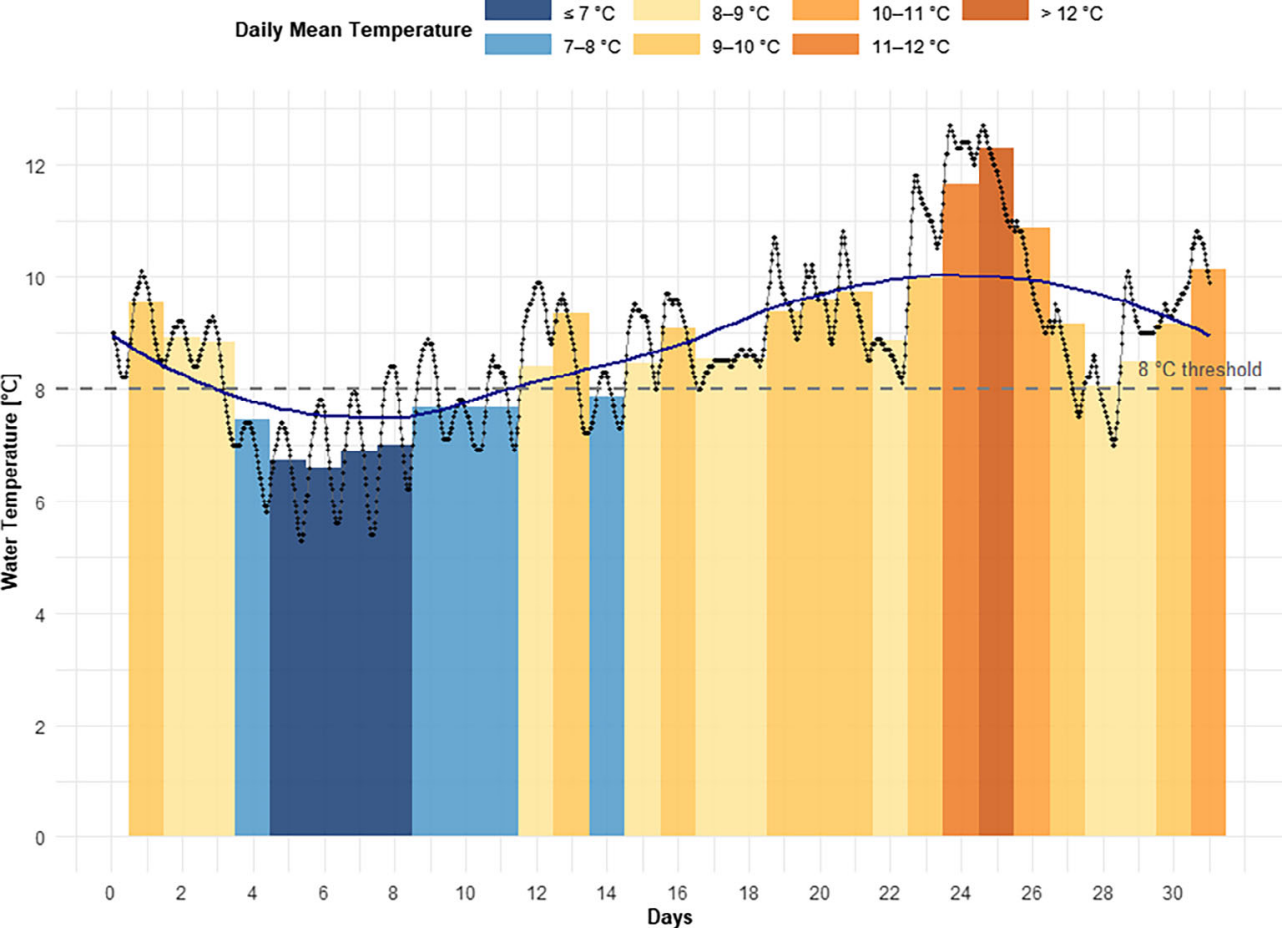
Daily mean water temperatures (bars, colour coded by temperature range) and individual temperature measurements (points) with LOESS (locally weighted scatterplot smoothing, blue line) of the River Ilme for 31 days (30 March 2023 to 29 April 2023). The dashed horizontal line indicates the 8°C threshold. Colour classes reflect specific temperature intervals, facilitating interpretation of thermal conditions during early embryonic development.

With the onset of reproduction (30 March 2023, day 0), the water temperature of the Ilme was monitored (Figure 3). The length of the data series was adjusted and stopped with 264 DD (day 31), according to the results of the incubation experiment (see Section 3.1). The minimum water temperature was 5.3°C, and the maximum temperature reached 12.7°C. Over the period of record, the median water temperature of the river was 8.7°C. During early embryogenesis, wild grayling eggs might have been predominantly exposed to low water temperatures, whereas during later developmental stages, mean daily temperatures exceeded 12°C.

### 3.3 Body parameters

**FIGURE 4:**
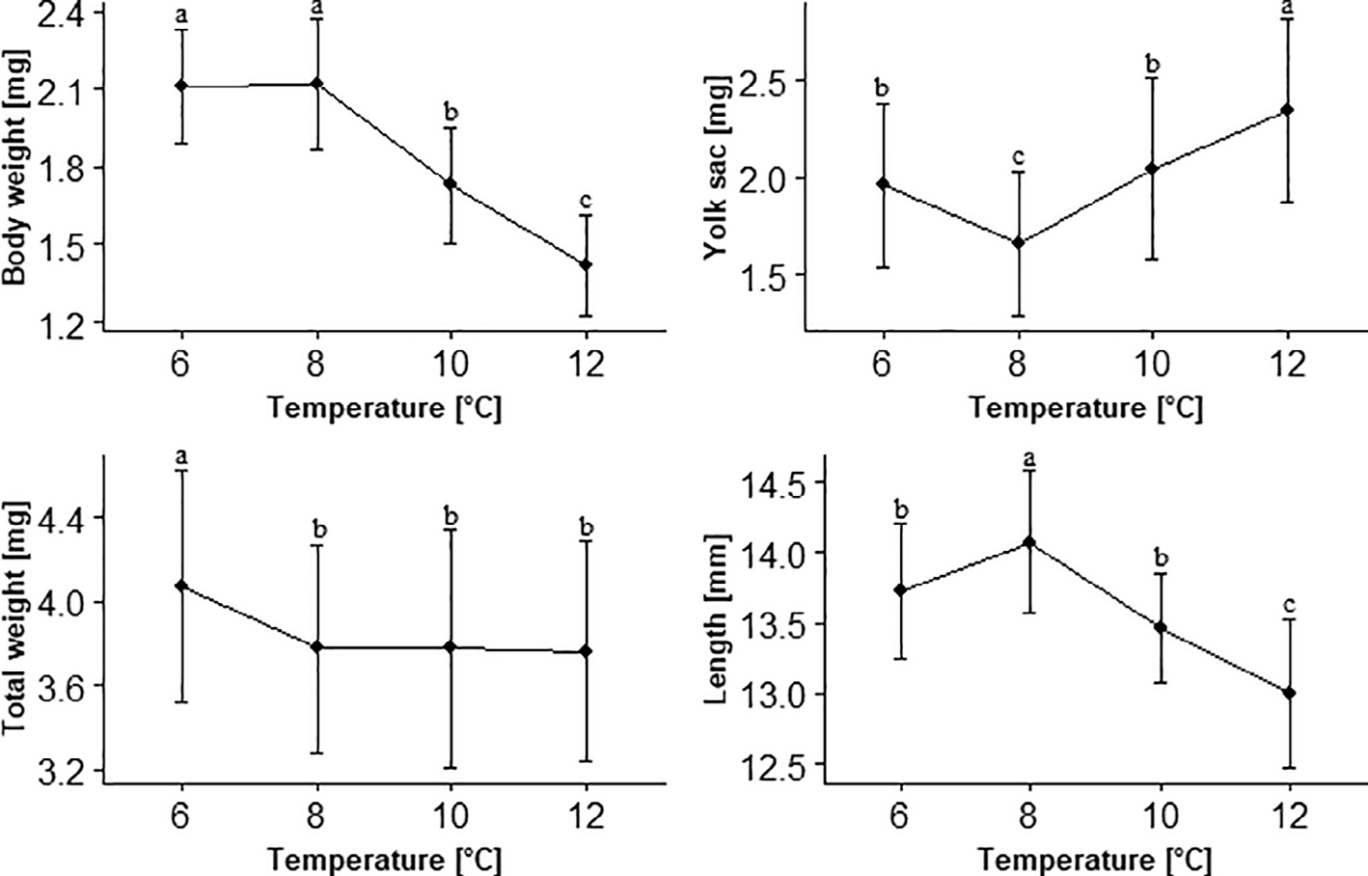
Body parameters of grayling larvae (*n* = 45 per treatment) incubated at four different temperatures. Weight parameters were measured as dry mass. Different letters in the same plot are significantly different at *p* < 0.05 (Tukey's test).

The biometric measures (weight and length) of the grayling larvae in response to varied incubation temperatures were recorded (Figure 4). The biometric measures (weight and length) of the grayling larvae in response to varied incubation temperatures were recorded (Figure 4). The highest larvae weight was observed in the 6 and 8°C groups, with a significant decrease in weight with increasing incubation temperatures (*p* < 0.05). Larvae had the lowest yolk‐sac weight at 8°C and the highest at 12°C (*p* < 0.05). Overall, the body weight did not differ between the 8, 10, and 12°C groups (*p* > 0.05) but was highest in the 6°C group (*p* < 0.05). The different incubation temperatures also changed the final length of the fish. Fish incubated at 8°C water temperature had the highest length, whereas it decreased with increasing and decreasing temperatures, respectively (*p* < 0.05).

## 4 DISCUSSION

The spawning of European grayling occurs from the end of March (Parkinson et al., 1999; Schmall, 2009) until mid‐June and is strongly influenced by the breeding ground observed (Eloranta, 1985; Kristiansen & Døving, 1996; Lahnsteiner & Kletzl, 2012; Linløkken, 1995; Scott, 1985), following the pattern that grayling in northern parts of its natural range tend to spawn later in the year. In addition, reproduction can be influenced by climate conditions (Lahnsteiner & Kletzl, 2012; Ovidio et al., 2004).

According to our experience through the establishment of a grayling population in aquaculture, the beginning of natural reproduction of this population is early March to early April in the studied area (River Ilme in Lower Saxony). This observation is in accordance with the literature, which indicates the time span for spawning to be 3–4 weeks (Charles et al., 2006; Wedekind & Küng, 2010). The recorded temperature profile of the River Ilme aligns with previously published data and corresponds to reproduction temperatures observed in other natural waters from 4 to 14°C (Ovidio et al., 2004; Scott, 1985; Witkowski & Kowalewski, 1988). However, limited historical data are available for the River Ilme and as such limits our ability to compare the long‐term effects of climate change. Although grayling from aquaculture share the same genetic origin and the same source of water, it has been observed that they reach spawning maturity 2–4 weeks later than the wild population. This phenomenon, previously described by Lahnsteiner and Kletzl (2012), may be attributed to suboptimal husbandry conditions affecting gamete maturation. Captivity has been shown to affect the reproductive traits of salmonids (Araki et al., 2007), and this has recently been confirmed in >3‐year‐old pond‐reared grayling (Avramović et al., 2024).

The duration of incubation to hatching has so far often been recorded with the help of observations in wild populations and varies strongly between literature sources. Reported incubation times vary between populations from 177 to 320 DD (Bašić et al., 2018; Cove et al., 2019; Humpesch, 1985; van Leeuwen et al., 2017). Therefore, it must also be noted that these studies do not distinguish between the start and completion of hatching. In this respect, the presented study provides a more detailed insight into embryonic development of grayling. Under a controlled environment the eyeing stage was reached after 96–108 DD, with the first‐hatched larvae observed between 152 and 168 DD, and at 228–264 DD all hatchings were complete.

First‐time spawners are known to exhibit a more pronounced higher survival rate of heavier grayling eggs; however, this effect diminishes over repeated spawners (Kodela et al., 2023), which are used in this study. The highest hatching rates were observed at 6 and 8°C treatment. With a survival rate of some clutches exceeding 85%, this study reported the highest survival rates to date for aquaculture‐reared grayling, compared to previous studies (Kodela et al., 2023; Szmyt et al., 2012; Szmyt et al., 2015).

Several indicators support the results of the highest hatching rate of larvae at 8°C. Firstly, the water temperature in the spawning area shows that even under natural conditions this population experiences ~8°C water temperature in this period. This observation is important for further small‐scale breeding projects, because the optimal incubation temperature could also be higher or lower due to the adaptation of populations to their local environments (Jensen et al., 2008). Therefore, it makes sense to know the condition of the natural environment. Secondly, a visually increased occurrence of fungal infections on the eggs was observed at higher incubation temperatures. Although this finding is based on qualitative observation, it highlights a potential temperature‐dependent vulnerability, as *Saprolegnia* infections are known to occur when water temperatures are suboptimal (Lindholm‐Lehto & Pylkkö, 2024). Thirdly, the results on body parameters indicate that the highest retention of the yolk‐sac and highest body weight and length is also achieved at 8°C. Higher incubation temperatures can induce early hatching (Crichigno et al., 2021; From & Rasmussen, 1991), meaning that the larvae must use up a larger part of the energy reserve outside of the egg. The larvae therefore have a lower body weight and reduced length as a result, but the yolk sac is heavier. This observation is in accordance with other studies for brown trout (*Salmo trutta*) and rainbow trout (*Oncorhynchus mykiss*) (Crichigno et al., 2021; Ojanguren & Braña, 2003). A similar result was obtained by Haugen and Vollestad (2000) but only in one of the populations studied. The other two populations exhibited a contradictory effect. However, differences between populations may arise not only due to geographical distance but also as a result of local adaptation to environmental conditions such as temperature regimes. Such adaptations often correspond to genetic differentiation between populations inhabiting distinct river systems (Haugen & Vollestad, 2000).

The only study with controlled temperature regimes was conducted by Jungwirth and Winkler (1984), who investigated incubation temperatures ranging from 2 to 16°C. Contrary to our study, the results showed that the optimal incubation temperature varies widely between 6 and 13.5°C. It should be noted that this study was conducted with only 200 grayling eggs per treatment. In contrast, our experiments included a minimum of 300 and up to >1500 eggs per treatment, providing a more robust dataset for assessing temperature effects. The use of wild‐caught grayling by the authors may influence egg quality. In the present study, one clutch exhibited a pronounced red colouration, resembling that of wild grayling eggs, and achieved high hatching rates across all treatments. These elevated hatching rates may be linked to increased egg quality, which in turn could result from the female's consumption of natural prey. Egg quality is largely determined by the nutritional composition of the yolk, which reflects the maternal diet prior to spawning (Haga et al., 2008). Because the fish are kept in an earthen flow‐through pond with relatively low stocking density and fed a commercial diet without carotenoids, the female might be feeding on natural feed such as *Gammarus* spp., which is known to contain high amounts of carotenoids (Babin et al., 2020; Czeczuga, 1980). The freshwater amphipods were sighted during the experiment when the grayling were caught and are typical of German river systems (Thepphachanh & Stamm, 2023). Carotenoids like astaxanthin have a positive effect on reproduction traits in salmonid fish (Ahmadi et al., 2006; Elbahnaswy & Elshopakey, 2023) and can significantly increase survival rates (Tyndale et al., 2008). This could explain to some extent the results, but so far it is not known whether a higher carotenoid content in the egg also increases temperature tolerance during incubation. For this reason, a nutritional influence cannot be ruled out. On the contrary, genetic factors, at least on the paternal side, can be excluded using a sperm mix. Therefore, it is reasonable to assume that there is a nutritional influence, but further investigations are necessary to understand these observations on egg and embryo survival and thermal tolerance.

The presented study and Charles et al. (2006) suggest a strong effect of temperature on grayling populations. The data suggest that the reproductive capacity of grayling decreases as water temperatures increase, likely due to increased embryonic mortality. This highlights the potential negative impact of climate change on the fertility of grayling populations. In addition, the increasing number of extreme weather events may lead to short‐term higher water temperatures, which will lead to a lower hatching rate. However, an additional effect is that the spawning period can be postponed due to increasing water temperatures in spring. Wedekind and Küng (2010) demonstrated that increasing water temperatures over a 60‐year period resulted in an earlier spawning time by 3–4 weeks. Consequently, larvae develop in colder environments due to earlier reproduction, which may cause issues with the availability of zooplankton as an initial food source.

## 5 CONCLUSION

Breeding grayling is important, especially with regard to the growing need to restock declining waters, which helps maintain the vitality of fishing areas and ensures the long‐term sustainability of fish populations. This study shows that species conservation projects involving artificial reproduction of fish should be oriented towards the natural temperatures in the river of origin. Therefore, it is essential that long‐term data from the reproduction site are utilized. For the grayling population studied, an incubation temperature of 6 and 8°C is optimal. However, incubation temperature optima may vary between populations, particularly in response to local adaptation and genetic differentiation, especially when populations are geographically distant or inhabit distinct river systems. Results indicate that there might be an additional effect of feed and carotenoid content of the eggs on the survival at increased incubation temperatures and should be considered in future research. Overall, the findings show that successful propagation of grayling is possible under good aquaculture practices and high hatching rates are achievable.

## AUTHOR CONTRIBUTIONS

**Simon Rosenau:** conceptualization, methodology, visualization, formal analysis, investigation, writing – original draft, project administration. **Christian Lodder:** methodology. **Alexander Charles Mott:** writing – review and editing. **Jens Tetens:** resources, writing – review and editing, supervision.

## CONFLICT OF INTEREST STATEMENT

The authors declare that they have no known financial or personal conflicts of interest that could have influenced the reported work.

## FUNDING INFORMATION

The article was funded by Open Access Publication Funds of the Göttingen University.

## Supporting information


**Data S1.** Supporting information.
