# Supplementary material for: Potential impact of climate change on the reproductive success of grayling ( Thymallus thymallus )
Source: J Fish Biol. 2025 Aug 14;107(5):1778–87. doi: 10.1111/jfb.70160 (PMC12710855; doi:10.1111/jfb.70160)
Supplement: Supplementary file 1 — Data S1. Supporting information. [file JFB-107-1778-s001.docx]

1. Supplementals

*Supplementary Table* *1*. Total number of eggs per clutch and treatment across four temperature treatments (6 – 12 °C).

| **Treatment** | **Clutch** | | | | | | | | | **Total eggs per treatment** |
| --- | --- | --- | --- | --- | --- | --- | --- | --- | --- | --- |
|  | 1 | 2 | 3 | 4 | 5 | 6 | 7 | 8 | 9 |  |
| 6 °C | 1526 | 1157 | 843 | 785 | 919 | 705 | 490 | 399 | 317 | 7141 |
| 8 °C | 1467 | 1184 | 863 | 800 | 911 | 742 | 470 | 342 | 311 | 7090 |
| 10 °C | 1536 | 1176 | 895 | 807 | 955 | 756 | 524 | 389 | 307 | 7345 |
| 12 °C | 1375 | 1274 | 935 | 838 | 905 | 754 | 565 | 355 | 329 | 7330 |
|  |  |  |  |  |  |  |  |  |  |  |


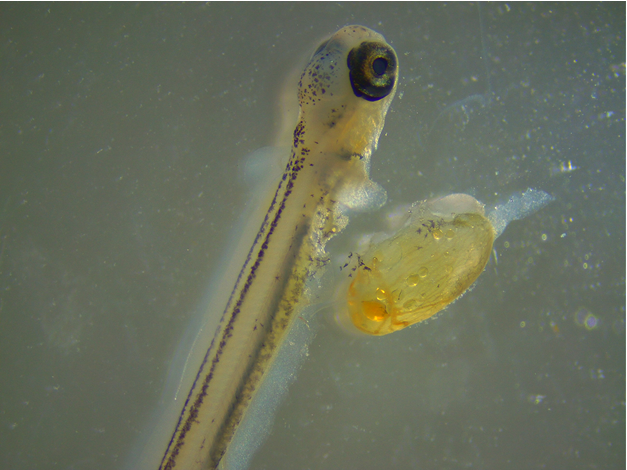


*Supplementary Figure* *1*. Photo of a grayling larvae with a dissected yolk sac.

*Supplementary Table* *2*. Percentage mortality rate of hatched larvae (calculated as a proportion of the total number of eggs) from nine clutches incubated under four different water temperatures (6 – 12 °C).

| **Treatment** | **Clutch** | | | | | | | | |  |
| --- | --- | --- | --- | --- | --- | --- | --- | --- | --- | --- |
|  | **1** | **2** | **3** | **4** | **5** | **6** | **7** | **8** | **9** | **Mean ± SD** |
| 6 °C | 0.92% | 9.25% | 3.08% | 2.17% | 2.29% | 2.84% | 4.29% | 1.25% | 4.73% | 3.42 ± 2.52% |
| 8 °C | 2.18% | 7.35% | 2.43% | 1.38% | 1.54% | 4.31% | 1.28% | 0.58% | 0.96% | 2.45 ± 2.14% |
| 10 °C | 3.65% | 6.46% | 8.94% | 6.20% | 1.78% | 6.22% | 8.59% | 6.68% | 3.91% | 5.82 ± 2.33% |
| 12 °C | 5.53% | 0.31% | 11.44% | 3.70% | 3.65% | 9.28% | 4.60% | 3.38% | 4.86% | 5.20 ± 3.32% |

*Supplementary Table* *3*. Percentage survival rate (calculated as a proportion of the total number of eggs) of nine clutches incubated under four different water temperatures (6 – 12 °C).

| **Treatment** | **Clutch** | | | | | | | | |  |
| --- | --- | --- | --- | --- | --- | --- | --- | --- | --- | --- |
|  | **1** | **2** | **3** | **4** | **5** | **6** | **7** | **8** | **9** | **Mean ± SD** |
| 6 °C | 83.88% | 69.58% | 76.28% | 80.25% | 86.07% | 58.87% | 79.59% | 83.21% | 74.13% | 76.87 ± 8.01% |
| 8 °C | 86.64% | 75.34% | 84.01% | 74.50% | 85.73% | 66.98% | 81.70% | 85.96% | 82.32% | 80.35 ± 6.30% |
| 10 °C | 82.88% | 44.47% | 70.73% | 58.36% | 85.65% | 62.04% | 52.67% | 79.18% | 80.13% | 68.46 ± 13.85% |
| 12 °C | 64.80% | 0.94% | 51.87% | 3.46% | 77.57% | 39.39% | 53.27% | 81.13% | 37.39% | 45.54 ± 27.14% |
